# Optical coherence tomography assessment of a complex bifurcation lesion treated with double kissing Crush technique: A case report

**DOI:** 10.1097/MD.0000000000005740

**Published:** 2017-01-10

**Authors:** Jin-Zan Cai, Yao-Jun Zhang, Tian Xu, Yong-Xiang Zhu, Chen-Yu Mao, Christos V. Bourantas, Tom Crake, Shao-Liang Chen

**Affiliations:** a Department of Cardiology, Nanjing First Hospital, Nanjing Medical University, Nanjing, China; b Department of Cardiovascular Sciences, University College London; c Department of Cardiology, Barts Heart Centre, London, UK.

**Keywords:** coronary bifurcation lesion, double kissing Crush, optical coherence tomography

## Abstract

The DEFINITION (Impact of the complexity of bifurcation lesions treated with drug-eluting stents) study has provided a novel classification to evaluate the complexity of coronary bifurcation lesion according to coronary angiography, but angiographic imaging due to its low resolution and inherited limitation may result in an inaccurate adjudication.

We used optical coherence tomography (OCT) to further evaluate the coronary characteristics in a patient with “simple” bifurcation lesion which was classified by the DEFINITION criteria. However, a “complex” bifurcation lesion was defined and confirmed according to the OCT results.

A double kissing Crush stenting approach was adopted to treat this “complex” case finally. The immediate and long-term angiographic and OCT results were excellent.

OCT may be useful imaging modality to classify complexity of coronary bifurcation lesion and subsequently guide its treatment strategy.

## Abstract 2

Supplemental Digital Content is available in the text

## 1 Introduction

The treatment of coronary bifurcation lesions can be challenging and associated with a high incidence of major adverse cardiac events. The Consensus of the European Bifurcation Club recommends provisional stenting as the preferred technique for the majority of bifurcation lesions.^[1]^ However, a single stent strategy may lead to acute side branch occlusion, suboptimal angiographic results with a residual stenosis in the side branch, side branch restenosis, and the need for re-intervention at follow-up. The DEFINITION (Impact of the complexity of bifurcation lesions treated with drug-eluting stents) study has provided evidence that complex bifurcation stenting—and in particular double kissing (DK) Crush technique—is associated with better clinical outcomes in lesions with a specific anatomical characteristics assessed by coronary angiography.^[2]^ However, coronary angiography may often fail to evaluate coronary artery pathology. In this case report, we used for the first time optical coherence tomography (OCT)—rather than coronary angiography—to classify lesion severity according to the DEFINITION criteria and assess the immediate and long term results of a DK Crush stenting.

## 2 Case report

A 50-year-old male patient with a history of hypertension, current smoker, and hyperlipidemia was admitted with angina symptoms that appeared 2 weeks ago. There was no positive finding from the relevant physical examination. Electrocardiogram demonstrated ST-segment elevations in leads V1 to V4, and the troponin was raised. The patient diagnosed with an acute myocardial infarction was treated conservatively in the local hospital and transferred to our center for coronary angiography/percutaneous coronary intervention (PCI).

**Figure 1:**
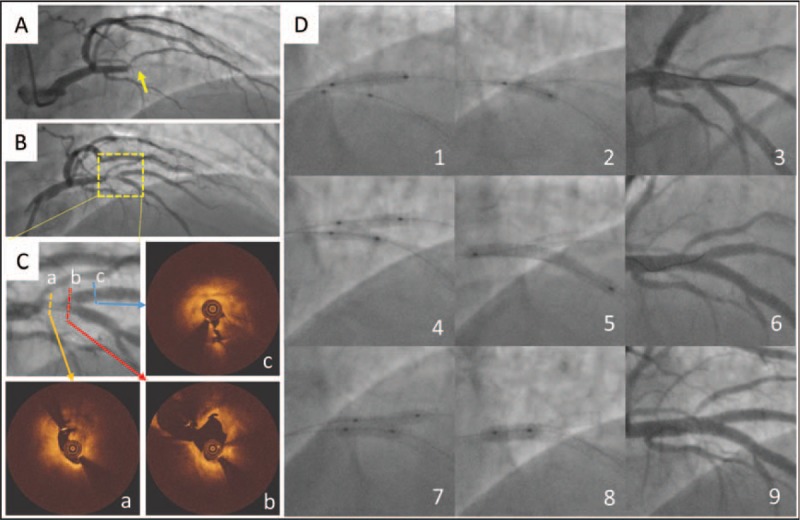
Stenting procedure for the thrombotic bifurcation lesion with double kissing Crush technique. (A) Coronary angiography showed a thrombotic occlusion in proximal LAD (arrowhead). (B, C) Coronary angiography revealed a “complex” bifurcation lesion on LAD/diagonal 2, which was also confirmed by OCT (a–c) after balloon predilation. (D) Stenting procedure for the bifurcation lesion with double kissing Crush technique (1–9). LAD = left anterior descending artery, OCT = optical coherence tomography.

Coronary angiography showed a thrombotic occlusion in the proximal left anterior descending (LAD) artery (Fig. 1). The lesion in the LAD artery was crossed with a hydrophilic guide-wire and dilated with a 2.5 × 15 mm compliant balloon at 10 atm. Repeat angiography revealed a LAD/second diagonal (D2) “simple” bifurcation lesion classified by the DEFINITION criteria. However, a more detailed assessment of lesion anatomy with OCT imaging at the main and the side branch indicated a “complex” bifurcation lesion according to the DEFINITION classification (main criteria: side branch diameter stenosis ≥90% and lesion length ≥10 mm, minor criteria: thrombus containing lesion and main vessel lesion length ≥25 mm) as there was thrombus and that caused a significant obstruction not only in the main but also in the side branch.

**Figure 2:**
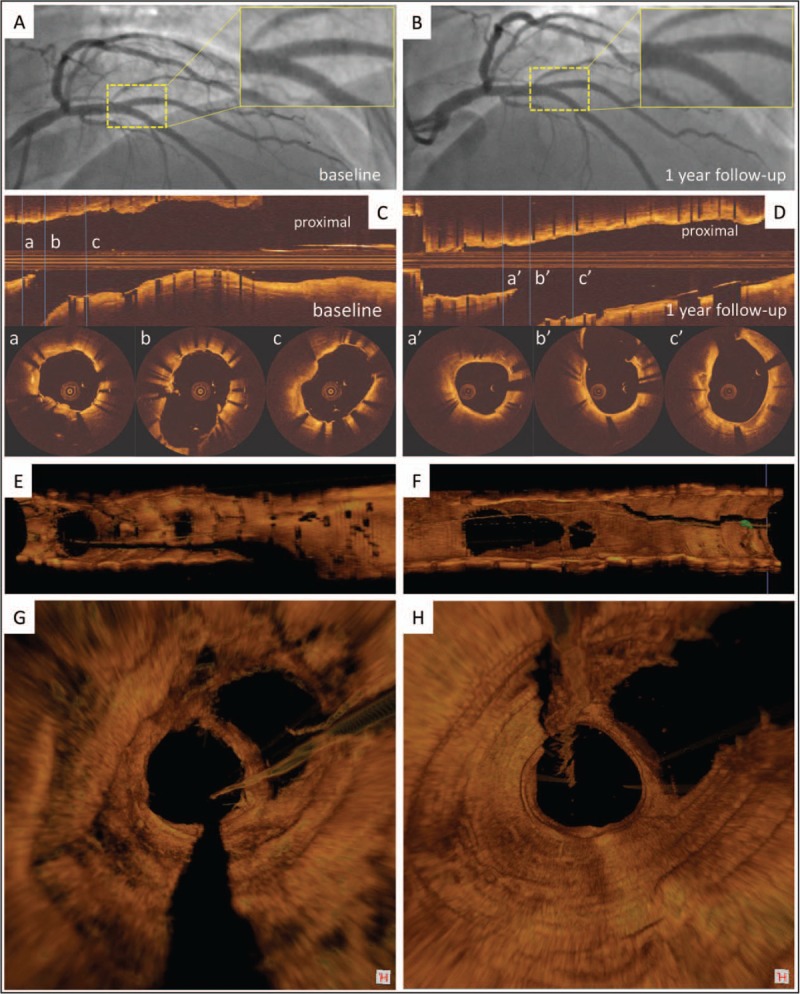
The results of angiography and OCT post-procedure and at 1-year follow-up. (A) Final angiography after stenting the bifurcation lesion. (B) Angiography at 1-year follow-up. (C) OCT longitudinal views postprocedure and (D) at 1-year follow-up, together with cross-sectional views (a–c and a’–c’). (E–H) Three-dimensional OCT reconstruction confirmed no malapposed stent struts in bifurcation carina and POC area postprocedure. (F–I) The stent struts in bifurcation carina and POC area were well covered by a thin neointimal hyperplasia at 1-year follow-up. OCT = optical coherence tomography, POC = polygon of confluence.

A 2.75 × 33 mm drug-eluting stent was implanted in the mid-LAD artery before a DK Crush stenting approach adopted to treat this “complex” case (see Fig. 1, Supplemental Digital Content, which showed the stent was fully expanded without flow restriction or dissection). First, a 2.75 × 14 mm drug-eluting stent was implanted in the D2 with 3 mm protruding in the main vessel (Fig. 1, D1). The protruded segment was crushed by a noncompliant balloon (3.0 × 12 mm) (Fig. 1, D2). The deployed stent in D2 was re-wired in which the wire position was confirmed by orthogonal projections and the ostium of SB was then dilated with a noncompliant balloon (2.75 × 14 mm) (Fig. 1, D3). Kissing balloon inflation was performed with a 2.75 × 20 mm noncompliant balloon in D2 and a 3.0 × 14 mm noncompliant balloon in LAD artery (Fig. 1, D4). Then, a 3.0 × 33 mm drug-eluting stent was deployed in the proximal LAD artery followed by postdilation (Fig. 1, D5). The stent in the D2 was re-wired through the proximal/middle stent cell of the stent in the LAD artery (Fig. 1, D6). Kissing balloon was performed using a 2.75 × 12 mm and 3.0 × 12 mm noncompliant balloons at 10 atm (Fig. 1, D7), and then proximal optimization technique was conducted with a 3.5 × 9 mm noncompliant balloon (Fig. 1, D8 and D9). Coronary angiography demonstrated an excellent angiographic result. OCT examination in the LAD artery and D2 demonstrated complete stent expansion at the main vessel and the ostium of the D2 with only a few malapposed stent struts in the most proximal LAD artery. Offline 3-dimensional OCT reconstruction confirmed no malapposed stent struts in the carina of the bifurcation and polygon of confluence area (Fig. 2).

At 1-year follow-up, no major adverse cardiac events were reported. Angiographic examination showed no obvious lumen obstruction within the stented segment. OCT and offline 3-dimensional reconstruction demonstrated that stent struts in both LAD and D2 were well covered by a thin neointimal layer that did not cause significant obstruction (Fig. 2).

## 3 Discussion

The DEFINITION classification for bifurcation lesions provides a practically useful solution to differentiate “simple” and “complex” bifurcation lesions and subsequently help for treatment strategy selection. However, DEFINITION relies on coronary angiography, which quite often fails to accurately assess lesion anatomy and severity.^[2]^ OCT can provide unique insights in this setting enabling detailed evaluation of coronary bifurcation pathology and facilitating procedural planning.^[3]^ This is the first case in which we implemented OCT imaging to examine coronary artery pathology and use the DEFINITION classification to guide treatment of a bifurcation lesion. OCT imaging did not only permit detection of significant disease and thrombus at the ostium of the diagonal that was not seen by coronary angiography but also provided useful anatomical information and detailed assessment of the reference luminal dimensions and of the length of the lesions that facilitated selection of the appropriate balloon and stent size. Repeat imaging poststent implantation allowed assessment of stent expansion and identification of malapposed struts providing unique information for further treatment planning. The 1-year follow-up imaging data demonstrated complete struts coverage even in segments with 3 layers of struts, and no evidence of instent restenosis.

In the past years, OCT imaging contributed tremendously to the optimization of bifurcation stenting techniques. With the high resolution, OCT could facilitate interventionlists to re-cross proper stent cell, which is the key procedure in both provisional stenting and 2-stent techniques.^[4–6]^ Moreover, poststenting OCT imaging provides unique information for further optimal treatment strategy. Burzotta et al^[7]^ have reported that poststenting OCT findings led to additional interventions in 16 of 55 bifurcation lesions (29.1%) which presented stent strut malapposition, major dissection, major tissue prolapsed, or intracoronary thrombus. Nevertheless, there are limited data about the clinical value of OCT in guiding PCI. Although recent registries have shown potential benefit of OCT guidance, there are no data in the context of randomized controls trials that would support its use in the clinical setting.^[8]^ Futures studies including the ILLUMINA III (NCT 2471586) and OPINION (NCT 01873027) are anticipated to provide further evidence about the clinical value of the OCT guiding PCI.

## Supplementary Material

Supplemental Digital Content
