# Supplementary material for: Optical coherence tomography assessment of a complex bifurcation lesion treated with double kissing Crush technique: A case report
Source: Medicine (Baltimore). 2017 Jan 10;96(1):e5740. doi: 10.1097/MD.0000000000005740 (PMC5228674; doi:10.1097/MD.0000000000005740)
Supplement: Supplemental Digital Content [file medi-96-e5740-s001.docx]

**Title:** Optical Coherence Tomography Assessment of a Complex Bifurcation Lesion Treated with Double Kissing Crush Technique: Case Report

**Authors:** Jin-Zan Cai, MSc;

**
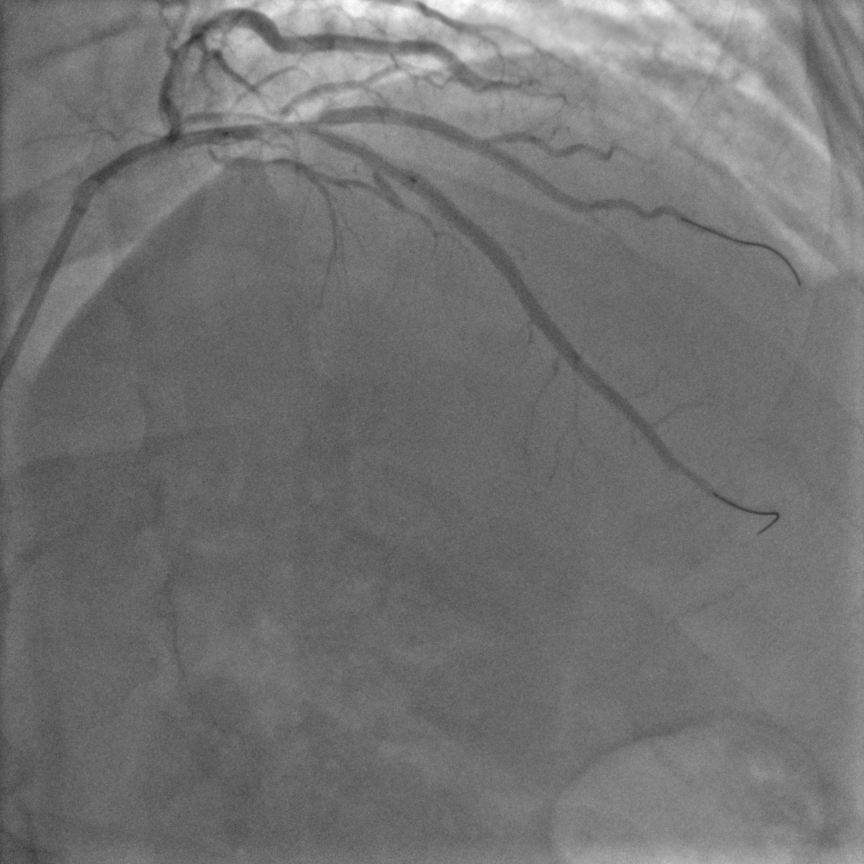
**

**Figure 1**

**Title:** The angiography after stent implantation in mid-LAD.

**Caption:** A 2.75*33 mm drug-eluting stent was implanted in the mid-LAD, and angiography showed that the stent was fully expanded without flow restriction or dissection.

**Abbreviation:** LAD=left anterior descending artery.
